# Pathologist-initiated whole genome and transcriptome sequencing demonstrates diagnostic utility in resolving difficult-to-diagnose tumors

**DOI:** 10.1186/s13073-025-01534-5

**Published:** 2025-10-07

**Authors:** Joseph H.A. Vissers, Catherine Mitchell, Owen W. J. Prall, Wing-Yee Lo, Sehrish Kanwal, Stephen J. Luen, Stephen C. Watts, Christopher M. Angel, Christine Khoo, Jia-Min B. Pang, William K. Murray, Cameron Snell, Michael Christie, Richard J. Rebello, Richard W. Tothill, Kym Pham, Oliver Hofmann, Stephen B. Fox, Sean M. Grimmond

**Affiliations:** 1 Collaborative Centre for Genomic Cancer Medicine, University of Melbourne, Melbourne, VIC Australia; 2 Department of Clinical Pathology, University of Melbourne, Melbourne, VIC Australia; 3 Department of Pathology, Peter MacCallum Cancer Centre, Melbourne, VIC Australia; 4 Sir Peter MacCallum Department of Oncology, University of Melbourne, Melbourne, VIC Australia; 5 Department of Medical Oncology, Peter MacCallum Cancer Centre, Melbourne, VIC Australia; 6 Department of Anatomical Pathology, NSW Health Pathology, John Hunter Hospital, New Lambton Heights, NSW Australia; 7 Department of Pathology, Royal Melbourne Hospital, Melbourne, VIC Australia

**Keywords:** Diagnostic histopathology, Diagnostic dilemma, Whole genome and transcriptome sequencing, Next generation sequencing, Tumor classification

## Abstract

**Background:**

Despite significant advances in diagnostic cancer histopathology, a subset of tumors are unable to be classified using WHO criteria. The resulting diagnostic uncertainty can result in inappropriate clinical management and negative patient outcomes.

**Methods:**

We investigated whether combining histopathology with whole genome and transcriptome sequencing (WGTS) could improve the classification of tumors that posed diagnostic dilemmas despite extensive histopathology and standard molecular work-up at a quaternary oncology center.

**Results:**

We successfully sequenced 45 tumors from an initial set of 54 unclassified tumors (83% success rate). A confident diagnosis was made for 35/45 tumors (78%). Additionally, potential treatment targets were identified in 21/45 tumors (47%). Theoretical comparison with alternative assays demonstrated that WGTS was uniquely capable of detecting critical diagnostic findings in 9/35 tumors (26%).

**Conclusions:**

This work supports augmenting histopathology and standard molecular pathology with WGTS in the classification of difficult-to-diagnose tumors.

**Supplementary Information:**

The online version contains supplementary material available at 10.1186/s13073-025-01534-5.

## Background

In oncology, as in other areas of medicine, accurate diagnosis is critical to guiding appropriate management. Diagnosis of a tumor is usually established via assessment of a tissue biopsy, with histopathologists responsible for classifying the tumor, using systems such as the WHO classification of tumors[1]. Tumor classification is traditionally based on morphology, centered on recognizing the differentiation of the tumor, i.e., the degree to which the tumor resembles normal tissues. For a subset of tumor biopsies, establishing a definitive diagnosis is challenging for a variety of reasons, including the undifferentiated nature of the tumor, ambiguous morphologic or immunophenotypic features, or discordance between clinical, morphologic, and ancillary testing findings. These “diagnostic dilemmas” remain unclassifiable with standard histopathological work-up, resulting in difficulty in determining the most appropriate management course for the patient.

In the era of precision medicine, histopathological diagnosis may be complemented by molecular testing to identify tumor driver mutations or other alterations, which may be utilized to guide therapy. However, the role of molecular testing extends beyond identification of therapeutic targets, with identification of tumor molecular alterations that are pathognomonic or highly supportive of a diagnosis[1]. Histopathologists are therefore utilizing “molecular” immunohistochemistry[2], fluorescence in-situ hybridization or targeted next-generation sequencing (NGS) panels in routine diagnostics[3].

The utility of incorporating molecular alterations as an augment to histopathological diagnosis has been established in a wide range of tumor types, including both undifferentiated malignancies[4, 5] and specific tumor types, such as renal cell carcinoma[6] and SWI/SNF complex-deficient neoplasms[7–9]. Molecular tumor profiling to aid cancer diagnosis and identify therapeutically actionable targets has been extensively applied in the context of cancer of unknown primary (CUP), defined as metastatic cancer derived from an unknown primary site of disease[3, 10–14]. However, beyond the site of origin of metastatic cancer, pathologists face diagnostic dilemmas extending to subtyping and the malignant potential of tumors.

Whole genome and transcriptome sequencing (WGTS) allows comprehensive analysis of a tumor, but limitations such as cost, availability, and complexity have restricted its use to the research setting. More recently, WGTS has been shown to be feasible in routine clinical practice, with utility for detecting potential therapeutic targets in patients with metastatic cancer[15] and high-risk pediatric cancers[16]. In combination with histopathology, WGTS has also been used to establish tumor diagnoses in specific clinical settings, including pediatric and young adult cancers[17], sarcoma[18–20], and metastatic cancer of unknown primary (CUP), including undifferentiated CUP[21–24].

The success of these studies led us to postulate that WGTS may also be able to assist in establishing a diagnosis in other clinical settings. In this study, we specifically examined the diagnostic utility of WGTS in adult tumors, where a likely primary site of origin was identified, but definitive tumor classification, lineage (e.g., carcinoma, sarcoma), or malignant potential remained elusive despite extensive conventional histopathological and molecular work-up. We used a model whereby pathologists, in close consultation with a genetic analyst, selected difficult-to-diagnose cases in routine cancer care. Treating clinicians facilitated patient consent and enrolment but were not involved in case selection.

## Methods

### Patient selection

All patients were recruited to the Cancer of Low survival and UnMet Need – Pathologist Initiated (COLUMN-PI) study during a 3-year period with informed consent under an approved protocol at the Royal Melbourne Hospital Human Research Ethics Committee (HREC Reference number: HREC/61352/MH-2020 34). No patient identifying information has been included in this study, and all patients are referenced with anonymous identifiers, where identities are only known to the study team.

We prospectively identified patients whose tumors posed diagnostic dilemmas following routine histopathological work-up at the Peter MacCallum Cancer Centre (Peter Mac), a quaternary referral center in Melbourne, Australia, from September 2020 until June 2023, including cases for which biopsy or surgical resection was performed on site, as well as cases reported externally and referred for second opinion. The cases were reviewed (CM, JHAV, OWJP) to ensure that they met the following criteria:

(1) Cases posed a diagnostic dilemma despite extensive work-up, with either multiple differential diagnoses considered or divergent opinions between pathologists. Diagnostic work-up included standard morphological assessment, immunohistochemistry, fluorescence in situ hybridization, and targeted NGS panels if indicated and available. In addition, cases were reviewed by multiple histopathologists, including internal or external (both local and international) pathologists with expertise in the relevant field.

(2) The potential of WGTS to resolve the diagnosis on the basis of the expected genomic features for differential diagnoses.

(3) Resolving the diagnostic uncertainty predicted to impact management or prognosis.

(4) Tumors were either malignant or of uncertain malignant potential (tumors confidently recognized as benign were excluded).

(5) Patients were aged 18 years or older.

Following selection, the treating clinical team was consulted, and consent was then sought from the patients. Patients were excluded if they did not meet the criteria above, if there was insufficient tumor tissue available for testing, or if they were eligible for WGTS through an alternative program. The selected cases comprised approximately 0.2% of the cases reported during this time period.

Cases were reviewed by a histopathologist (CM, OWJP), and the nature of the diagnostic dilemma was quantified in several domains. First, we assigned a pre-WGTS morphologic diagnostic category on the basis of whether the tumor showed monomorphic (uniform) or pleomorphic (heterogeneous) cytology. Two subsets of cases were considered separately: one group included tumors with evidence of SWI/SNF deficiency (loss of expression of BRG1, encoded by *SMARCA4*, or INI1, encoded by *SMARCB1*) by immunohistochemistry, and the second group comprised renal epithelial neoplasms whose classification was difficult. Second, we recorded the number of potential lineages possible among diagnostic possibilities (e.g., epithelial, hematolymphoid, mesenchymal) and whether the malignant potential was known or uncertain.

### Whole genome and transcriptome sequencing

Fresh tissue biopsy specimens were collected and stored in “RNAlater” (Thermo Fisher, USA, cat. #AM7020) before DNA and RNA extraction using the Qiagen AllPrep DNA/RNA Mini Kit (Qiagen, USA, #80204). Otherwise, archived (FFPE) tissue specimens were used. Representative FFPE sections were reviewed by a pathologist, and tumor regions were macrodissected before nucleic acid extraction. Only FFPE tissue regions with > 30% tumor cellularity were selected for WGTS. DNA and RNA were extracted using the AllPrep DNA/RNA FFPE kit (QIAgen, USA, #80234). Whole blood samples were collected in either EDTA tubes (5 mL) or Streck DNA Blood Collection Tubes (Streck, USA) and extracted using the Qiagen QIAamp DNA Blood Mini Kit (Qiagen, USA, #51104).

FFPE-derived DNA was assessed for WGS suitability based on a modified *GAPDH *multiplex PCR assay that qualitatively estimates the proportion of non-overlapping DNA fragments of between 100 and 800 bp in a given sample[24, 25]. Samples were assigned scores of 1–8 depending on the largest amplifiable fragment (minimum “% Integrated Area” of 10%, as visualized by TapeStation 4200 D1000 electropherogram). Library preparation was attempted only on samples with a score of 4 or higher, indicating amplifiable fragments were 400 bp or longer.

DNA libraries were prepared using the Illumina TruSeq Nano library (Illumina, USA) method using 200 ng of DNA. All libraries were quality controlled using the TapeStation high sensitivity D5000 or D1000 ScreenTape (Agilent). Indexed libraries were pooled and sequenced aiming for a depth of 50× for normal and 100× for tumor using 150 bp paired reads on an Illumina NovaSeq 6000 platform (Illumina, USA).

WGS data were reprocessed as follows: primary and secondary analyses of WGS data were performed using the Illumina DRAGEN Bio-IT Platform v4.2.4[26]. Read data were aligned to the GRCh38 human reference genome with mapping, variant calling, and quality control completed using default settings. Tertiary analysis of WGS data (variant annotation and prioritization/filtering, fusion detection, mutational signatures, biomarker characterization) was carried out with sash v0.4.12[27]. WGS and WTS data were separately processed with oncoanalyser v2.0.0 [28] to obtain tissue of origin predictions from CUPPA[21] version 2.3.2[29].

TMB was calculated as outlined previously[30]. High TMB was defined as ≥ 10 mutations/Mb, and low TMB as < 10 mutations/Mb. COSMIC V2 base substitution mutational signatures[31, 32] were assigned using MutationalPatterns v3.8.0[33]. Dominant signatures were defined as the signature assigned to most somatic mutations in a case. Homologous recombination deficiency (HRD) was independently confirmed by HRDetect[34] and CHORD[35].

For WTS, RNA samples were subjected to ribosomal RNA depletion using the NEBNext rRNA Depletion kit (New England Biolabs) according to the manufacturer’s instructions. Approximately 100 million reads were generated per RNA sequencing library.

WTS data were aligned and quantified using the Illumina DRAGEN RNA pipeline (v4.2.4)[36]. Fusions were confirmed in RNA-seq data using Arriba v2.4.0[37, 38] and the DRAGEN fusion caller. Tertiary analysis of WTS data was performed using RNAsum, an R package that can post-process, summarize and visualize WTS data outputs[39].

Any additional information required to reanalyze the data reported in this paper is available from the lead contact upon request.

### Use of CUPPA

CUPPA (Cancer of Unknown Primary Prediction Algorithm) is a statistics-based tumor tissue of origin prediction tool trained on a reference set of > 7000 tumor samples[21]. We used CUPPA version 2.3.2, which utilizes both WGS and WTS data[29]. CUPPA results were only considered for cases with WGS-derived tumor cellularity estimates ≥ 30%. CUPPA predictions ≥ 80% were deemed high confidence[21, 24]. Cases where CUPPA predictions were the sole finding pointing towards a particular diagnosis were considered non-diagnostic.

### Diagnostic categories

Following WGTS, the molecular findings were discussed at an institutional molecular tumor board meeting, including the clinical team, curation scientists, and anatomical pathologists, and the final diagnosis resolved.

The cases were allocated a final diagnostic category by consensus (CM, JHAV, OWJP) according to the following criteria:

Category D1 = with pre-test data, WGTS strongly favors a specific diagnosis, per WHO classification or professional guidelines, or published in large/multiple reasonable/reputable papers or professional guidelines;

Category D2 = with pre-test data, WGTS resulted in an improved/reduced differential, resulting in diagnosis when considered with additional data (e.g., review of previous specimens, additional clinical information);

Category D3 = with pre-test data, WGTS supports a specific diagnosis, likely representing a newly characterized tumor type or subtype, on the basis of limited published studies but not included in the WHO classification;

Category D4 = with pre-test data, WGTS supports a specific diagnosis, favored to represent a novel tumor type or subtype, with limited published studies (case reports only), but not included in the WHO classification;

Category N1 = WGTS excluded one or more differential diagnoses, but definite diagnosis was not reached;

Category N2 = no/minimal change to diagnosis.

The cases were also re-reviewed by a histopathologist (CM, OWJP), and the number of potential lineages possible and malignant potential recorded again.

### Statistical tests

The chi-square test of independence was performed in Microsoft Excel to determine if outcomes were correlated with age < 50 years old, or monomorphic versus pleomorphic morphology. Details in Additional file 4.

### Methods for determination of potential therapeutic targets

Targeted therapy recommendations were based on public reimbursement guidelines and clinical trials available in Australia at the time of clinical reporting. All recommendations were endorsed by oncologists attending weekly institutional Molecular Tumor Board meetings.

### Retrospective review of potential clinical impact beyond diagnosis

Pathology reports, including histopathology and ancillary testing (e.g., FISH), and WGTS reports were reviewed by an experienced medical oncologist (SJL), who determined whether the WGTS findings were likely to impact the clinical management of the patient, considering the following:

1. Impact on prognosis, considering pre-WGTS diagnostic considerations and final diagnosis;
2. Impact on immediate management, particularly recommendations for adjuvant therapy;
3. Impact on future management in the potential setting of advanced disease; and
4. Impact on likely surveillance.

### Declaration of generative AI and AI-assisted technologies

During the preparation of this work, the authors used ChatGPT to improve the readability and language of the manuscript. After using this tool, the authors reviewed and edited the content as needed and take full responsibility for the content of the published article.

#### Figure generation

Oncoprint figures were generated using Oncoprinter[40–42]. Sankey plots were generated using SankeyMATIC[43]. The WGTS success rate plot was generated using R (v4.2) and webr package (v0.1.5). Figures were edited in Affinity Designer 2.

## Results

### Sample and patient characteristics

**Fig. 1:**
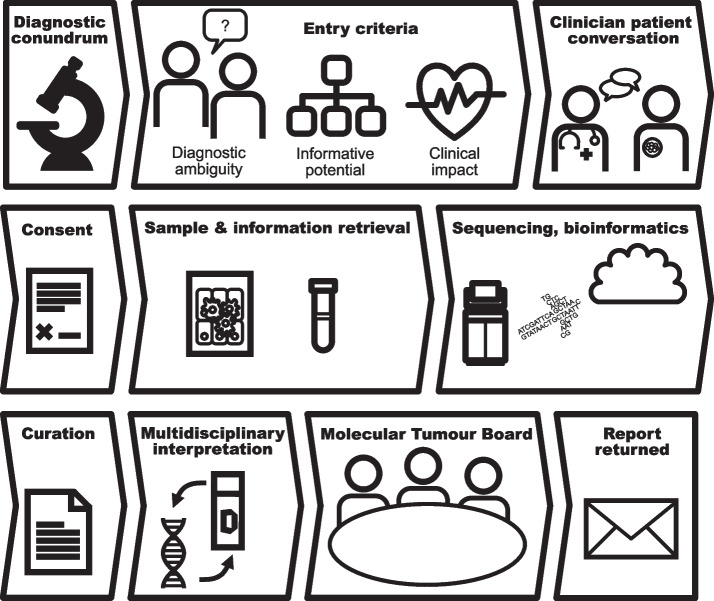
Work-flow schematic for the Pathologist-Initiated WGTS program

Figure 1 demonstrates the overall patient selection process and workflow for the study. We identified 54 patients who met eligibility criteria. Successful whole genome transcriptome sequencing (WGTS) was performed on 45 cases (83%) (Additional file 1: Fig S1). Nine cases failed quality metrics (DNA in four; sequencing in five). In 3 cases, multiple samples were sequenced (case 5: 3 samples, case 15: 2 samples and case 20: 2 samples). Low purity was the cause of fresh sample failure (2 of 15 samples, success rate 87%) (defined as no clinically significant findings could be detected). Poor-quality input material was the cause of failure of the FFPE-derived samples (4 of 38 samples, success rate 89%). The patient and sample characteristics of the cohort are described in Table 1.

**Table 1:** Patient and sample characteristics

Characteristic	No. of cases (*n* = 45)	%
**Age** (mean = 49, median = 49, range 19–81) years		
18–29	6	13%
30–39	11	24%
40–49	6	13%
50–59	7	16%
60–69	8	18%
70–79	6	13%
80 +	1	2%
**Gender**		
Female	25	56%
Male	20	44%
**Tissue type**		
Fresh tissue	14	31%
FFPE tissue	31	69%
**Biopsy site**		
Skin	6	13%
Head and neck	6	13%
Breast	4	9%
Lung, pleura, or mediastinum	5	11%
GI tract, liver, or peritoneum	4	9%
Kidney	7	16%
Lymph node	2	4%
Bone and soft tissue	11	24%
**Biopsy type**		
Small biopsy (core, endoscopic)	11	24%
Large biopsy (incision, excision)	34	76%
**Tumor primary or metastasis**		
Primary	24	53%
Metastasis	3	7%
Unclear	18	40%
**Possible recurrence of prior tumor**		
Yes	4	9%
No	41	91%

Work-up for each case included non-blinded review by at least two histopathologists with subspecialty expertise, as well as immunohistochemistry (mean number of antibody stains, 25; median, 23 per case), fluorescence in-situ hybridization (FISH, 16 cases), and targeted NGS panels (21 cases). Approximately half of the tumors (*n* = 24, 53%) were undifferentiated.

**Fig. 2:**
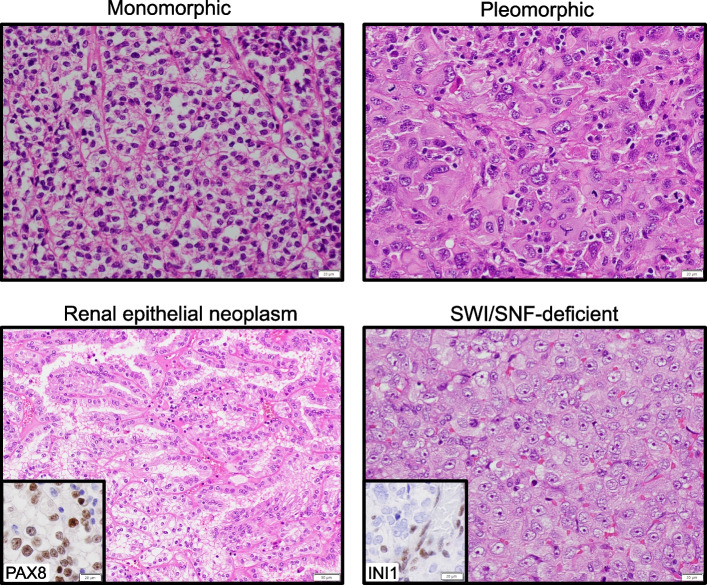
Categories of difficult-to-diagnose cancers Micrographs of H&E-stained slides representative of 4 categories of diagnostic dilemmas: Monomorphic, pleomorphic, renal epithelial neoplasm (inset: PAX8 immunohistochemical staining) and SWI/SNF-deficient (*SMARCB1-* or *SMARCA4*-deficient cases; inset shows INI1 staining; note the lack of staining in tumor cells compared with normal cells) categories

Prior to WGTS, tumors were classified into four groups (Fig. 2). In 18 cases, tumors were composed of a relatively uniform population of cells (designated “monomorphic”); this group included neoplasms with spindled, epithelioid, or round cell morphology, but, in an individual tumor, most cells exhibited a similar appearance. Another group of 16 tumors presented prominent variation in the appearance of tumor cells, designated “pleomorphic.” There were also seven difficult to classify “renal epithelial neoplasms,” and four “SWI/SNF-deficient” neoplasms with immunohistochemical loss of expression for either INI1 (encoded by *SMARCB1*) or BRG1 (encoded by *SMARCA4*). Overall, 24 cases were suspected to be primary, 18 either primary or metastatic and only 3 favored to be metastatic, i.e., for 93% of cases a primary tumor was considered probable or possible.

### WGTS findings

**Fig. 3:**
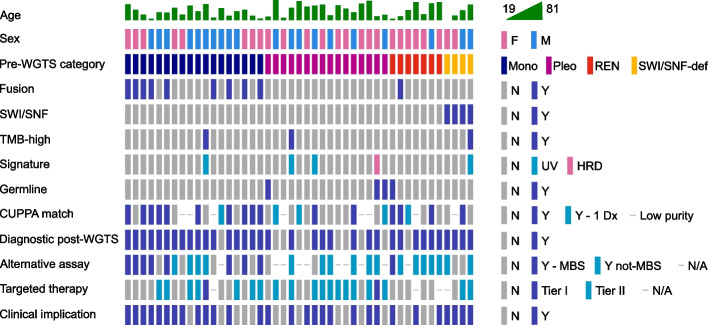
Oncoprint of 45 cases Sex: F – female; M – male. Pre-WGTS category: Mono – monomorphic; Pleo – pleomorphic; REN – Renal Epithelial Neoplasm; SWI/SNF-def – *SMARCB1* or *SMARCA4* deficient. Fusion: fusion gene detected. SWI/SNF: *SMARCB1* or *SMARCA4 *loss of functionmutations detected. Signature: mutational signature detected HRD – Homologous Recombination deficient; UV – Cosmic v2 signature 7 consistent with ultraviolet light-mediated DNA damage detected. Germline: reported germline variant. CUPPA match: N - No high confidence (> 80%) match; Y – High-confidence CUPPA match in agreement with human-inferred WGTS-informed diagnosis; Y - 1 Dx – High-confidence CUPPA match with one of multiple human-inferred differential diagnoses; Low purity – CUPPA not considered due to low tumor cellularity (<30%). Alternative assay: N – no alternative assay could have detected key diagnostic finding(s); Y- MBS – diagnostic finding could have been detected using assay reimbursed by the Medicare Benefits Schedule (Australia). Y not-MBS – diagnostic finding could have been detected using assay that is not reimbursed by the Medicare Benefits Schedule; N/A – not applicable owing to lack of diagnostic genetic alteration. Targeted therapy: N – no therapeutic target identified; Tier I – Target for National Comprehensive Cancer Network (NCCN) guideline-recommended, reimbursed treatment identified; Tier II – genetic alteration satisfying clinical trial entry criteria identified; N/A – not applicable owing to diagnosis of benign neoplasm. N – No; Y – Yes

WGTS findings are summarized in Fig. 3 and Additional files 2 and 3. Consistent with immunohistochemistry (IHC) results, *SMARCB1* (3 cases) or *SMARCA4* (1 case) loss-of-function mutations were detected in 4/4 SWI/SNF-deficient cases but not in any of the other three groups (0/41). Oncogenic gene fusions were detected in 10/45 (22%) cases. Tumors with monomorphic morphology were significantly enriched for fusions (9/18) compared with pleomorphic tumors (0/16, *p* = 0.001, Additional file 4). Mutational signatures associated with UV exposure were identified in 4/45 cases (9%, 1/18 monomorphic, 2/16 pleomorphic, 1/4 SWI/SNF-deficient), three had a high tumor mutational burden (≥ 10 mutations/Mb) and homologous recombination deficiency was detected in a single case (2%, 1/16 pleomorphic). Other useful diagnostic features included the finding of a near-haploid genome in 2/45 cases (4%) (Additional file 1: Fig S2a, b). Pathogenic germline cancer predisposition variants were present in 4/45 cases (9%); only one of these patients was known to carry a pathogenic germline variant prior to testing.

### Resolution of diagnosis

**Fig. 4:**
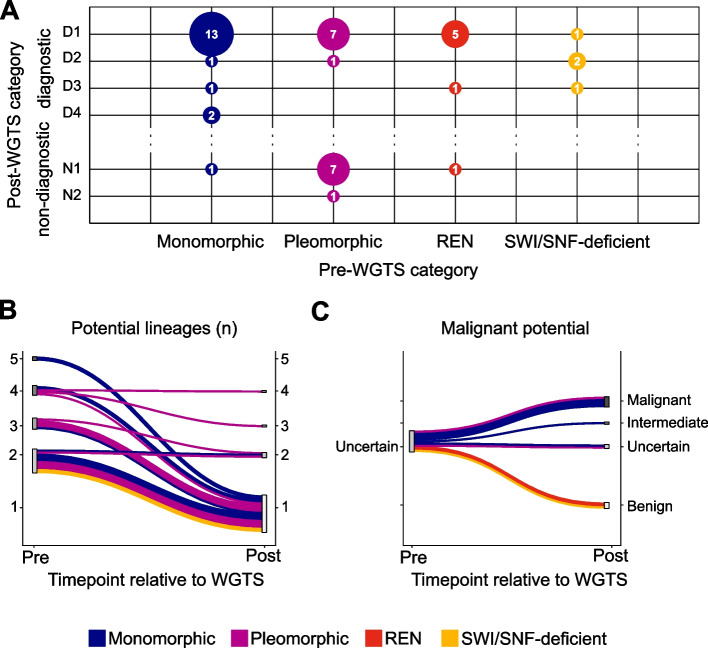
Diagnostic outcomes of WGTS **A** Bubble plot categorizing cases according to their pre-WGTS category (x-axis) and post-WGTS classification (y-axis). Bubble size is proportional to number of cases; numbers of cases are indicated in the center of the bubbles. Post-WGTS categories: D1 – diagnostic; pre-test data and WGTS strongly favor a specific diagnosis as per WHO guidelines, or published in large/multiple publications); D2 – diagnostic; pre-test data and WGTS not diagnostic but diagnostic based on additional follow-up data (e.g. previous specimens, clinician, external opinion, IHC staining); D3 – diagnostic; rare tumor type, currently published only in limited studies; D4 – diagnostic; likely novel tumor type or subtype; N1 – non-diagnostic, but improved differential (some diagnoses excluded); N2 – non-diagnostic, and no improvement in differential (i.e. no change). **B** Sankey plot illustrating the number of tumor lineages considered before and after WGTS. Only cases where multiple lineages were considered prior to WGTS are shown (*n*=25). Line thickness is proportional to the number of cases. Line color represents the pre-WGTS category. **C** Sankey plot illustrating the malignant potential of tumors considered before and after WGTS. Only cases with uncertain malignancy potential prior to WGTS are shown (*n*=11). Line thickness is proportional to the number of cases. Line color represents the pre-WGTS category

WGTS findings, in conjunction with clinical presentation, morphology, and immunophenotype, allowed a diagnosis to be established in 35 of 45 cases (78%), including 17/18 (94%) monomorphic, 8/16 (50%) pleomorphic, 6/7 (86%) renal epithelial, and 4/4 (100%) SWI/SNF-deficient tumors (Fig. 4a, D1–D4, representing diagnostic categories based on data sources used). Following WGTS, 36 were classified as primary tumors (80%) and 7 metastatic. There were 4 patients with a history of a prior malignancy for which a possible recurrence as an undifferentiated tumor was considered possible. In all 4 cases, WGTS findings were consistent with this.

**Fig. 5:**
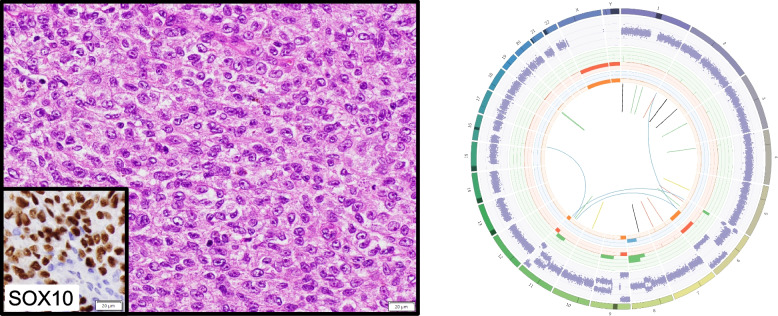
Example demonstrating utility of WGTS in resolving diagnosis Case 24. A 46-year-old man presented with a monomorphic epithelioid malignancy in the subcutis of the right gluteal region (photomicrograph of hematoxylin-and-eosin-stained section, left). Immunohistochemistry demonstrated positive expression of S100, SOX10 and Melan-A and retained expression of H3K27me3 and PRKAR1A (Sox10 immunohistochemistry, left inset). No disruption of EWSR1 was identified with break-apart FISH, and no variants were detected in BRAF, NRAS or KIT with a small targeted NGS panel. Based on these findings, a diagnosis of melanoma was favored, but the absence of cutaneous involvement, a previous cutaneous primary and a MAP kinase pathway abnormality were unusual. In this case, WGTS revealed GNAQ hotspot and SF3B1 missense mutations, and gain of chr8q including MYC (copy number 4) (Additional file 3). In combination with the subcutaneous location of the tumor, these findings are consistent with a diagnosis of melanoma arising within a blue nevus [63]. Importantly, a mutational signature consistent with UV exposure, which is frequently observed in cutaneous melanoma, was not detected. Thus, both presence and absence of features typically detected in diagnostic differentials were weighed to determine the most likely diagnosis (right) Tumor/normal WGS data are represented as a Circos plot. Note copy number gains (green) on chromosome 8q. Track 1: Chromosomes. Track 2: Beta Allele Frequency (allele frequencies of heterozygous SNPs that are common in germline samples). Track 3: Total copy number changes adjusted for tumor purity. Red = Loss; Green = Gain. Scaled from 0 (complete loss) to 6 (high-level gains). If > 6, shown as 6 with a green dot on the outermost green gridline. Track 4: Minor allele copy numbers. Range from 0 to 3. Expected normal minor allele copy number is 1, and anything below 1 is shown as a loss (Orange), representing an LOH (loss of heterozygosity) event. Minor allele copy numbers above 1 (Blue) indicate gains of both A and B alleles. Track 5 (Inner circle): Observed structural variants within or between the chromosomes. Blue = Translocations, Red = Deletions, Yellow = Insertions, Green = Tandem duplications, Black = Inversions

Among the 35 cases for which diagnosis was resolved, a diagnosis of a well-established tumor type was reached in 30 cases, i.e., a tumor type or subtype in the WHO Classification or equivalent, including 26 cases utilizing pre-test data in conjunction with WGTS findings [category D1] and four cases utilizing pre-test data, WGTS findings, and further information, such as review of previous biopsies [category D2]. For five additional cases, we identified emerging diagnoses not yet classified by the WHO[1] but supported by published small series of cases [category D3] or case studies [category D4]. An example demonstrating the utility of WGTS in resolving diagnosis is presented in Fig. 5 in a case of melanoma arising within a blue nevus.

Ten cases (22%) remained non-diagnostic (categories N1-N2), although in the majority of these (9/10 cases) some pre-WGTS differential diagnostic considerations could be eliminated (category N1, Fig. 4a). Tumors from younger patients were more likely to be successfully classified—only 2/23 cases from patients < 50 years old remained non-diagnostic compared with 8/22 cases from patients ≥ 50 years old (*p* = 0.03, Additional file 4).

CUPPA is a statistics-based tumor tissue of origin prediction tool trained on a reference set of > 7000 tumor WGS samples[21, 24]. Seventeen CUPPA predictions were concordant with our manual evaluation (Fig. 3). In five cases where WGTS was not able to resolve the diagnosis, CUPPA predicted a match with one of the differential diagnoses. For example, in case 15, we could not distinguish between histiocytic sarcoma and head and neck carcinoma, whereas CUPPA favored the latter. Overall, CUPPA lends independent support to our conclusions.

For the cases for which diagnosis was reached, we determined that use of one or more other assays could have potentially detected the diagnostic molecular findings in 26 of 35 cases (74%) without the need for WGTS (Fig. 3). This predominantly included targeted NGS panels, including small NGS panels such as the Thermofisher Oncomine Precision Assay (*n* = 5), large NGS panels such as the Illumina TruSight Oncology 500 panel (*n* = 11) and RNA fusion panel NGS such as the Illumina TruSight RNA Fusion Panel (*n* = 8), but occasional cases were potentially resolved with FISH (*n* = 1) or IHC (*n* = 1) alone (Additional file 2). We additionally predict that 19 of 35 cases (54%) could have theoretically been resolved through large panel NGS alone (Additional file 2).

### Resolution of tumor lineage and malignant potential

Although establishing a definitive diagnosis is ideal, clinical management can also be informed by identification of tumor lineage and/or malignant potential. For example, treatment pathways for sarcomas differ from those for carcinomas; post-operative clinical monitoring adheres to less rigorous criteria for benign neoplasms than for malignant neoplasms. Therefore, we also compared pre- and post-WGTS assessments of lineage and malignant potential.

Although epithelial lineage was known for the renal epithelial neoplasms, for 25 cases the pre-WGTS differential diagnoses included multiple tissue lineages (Fig. 4b), particularly in the monomorphic (10/18, 56%) and pleomorphic categories (13/16 cases, 81%). WGTS led to an overall reduction in the average number of potential lineages from 2.0 (pre-WGTS) to 1.2 (post-WGTS). For the 35 resolved cases, the post-WGTS lineages were mesenchymal (*n* = 15, 33%), epithelial (*n* = 15, 33%), melanocytic (*n* = 3, 7%), mesothelial (*n* = 1), and germ cell tumor (*n* = 1). Pre-WGTS 24 tumors were undifferentiated, and of these, a diagnosis was made in 17 cases (71%), with 6 remaining unresolved (29%). For 9/19 (53%) resolved undifferentiated cases, the lack of differentiation was typical for the tumor (e.g., sarcomas including cases 6, 7, and 11, or IDH-mutant sinonasal undifferentiated carcinoma, case 23), but 7/17 (41%) cases were resolved as dedifferentiated malignancies, including five carcinomas (one *SMARCA4*-deficient and one *SMARCB1*-deficient) and two sarcomas. Diagnoses were also made for three of five tumors showing transdifferentiation: a melanoma with chondroid differentiation (case 17), a malignant peripheral nerve sheath tumor with angiosarcomatous differentiation (case 30), and a metastatic uterine adenosarcoma with epithelioid morphology (case 32). The diagnostic utility of WGTS is highlighted by the resolution of these undifferentiated, dedifferentiated, and transdifferentiated cases, which are challenging with routine histopathology.

Prior to WGTS, 34 cases (76%) were interpreted as malignant on the basis of histological features, and in all cases, WGTS findings were consistent with malignancy (Additional file 2). In 11 other cases (24%), the pre-WGTS malignant potential was uncertain. WGTS resolved nine of these cases (82%), three of which were determined to be benign, one of intermediate malignant potential, and five as malignant (Fig. 4c). Importantly, two benign tumors were of renal origin, validating our approach in the renal epithelial neoplasm subset of cases where tissue of origin and lineage were already established. An example of a benign renal oncocytoma with a wholly diploid genome and chr11q13 rearrangement is presented in Supplementary Fig. 2c (Additional file 1). The remaining two cases remained of uncertain malignant potential, but WGTS supported neoplastic, rather than reactive, etiology.

### Value of WGTS beyond diagnostic utility

Identification of precision treatment targets is a key goal of cancer genomics. Potentially targetable molecular alterations were identified in 21/45 cases (47%) (Fig. 3, Additional file 1: Fig S3, Additional file 2). Clinical follow-up of each patient was beyond the scope of this project; however, review by an experienced medical oncologist suggested that WGTS provided additional information potentially useful for determining prognosis in 19/45 cases (42%), directing adjuvant therapy in 11/45 cases (24%), and directing therapy for advanced disease in 22/45 cases (49%) (Additional file 1: Fig S3). There were also potential impacts on surveillance intervals (6/45 cases, 13%). Overall, there was potential clinical utility beyond diagnosis in 30/45 cases (67%) (Fig. 3, Additional file 1: Fig S3).

In addition, variants predicting hereditary cancer predisposition syndromes were detected in the germlines of 4 patients (9%), with potential benefit for patients and their families (Fig. 3). All germline variants were detected in tumors from patients < 50 years old (4/23; *p* = 0.04, Additional file 4).

**Fig. 6:**
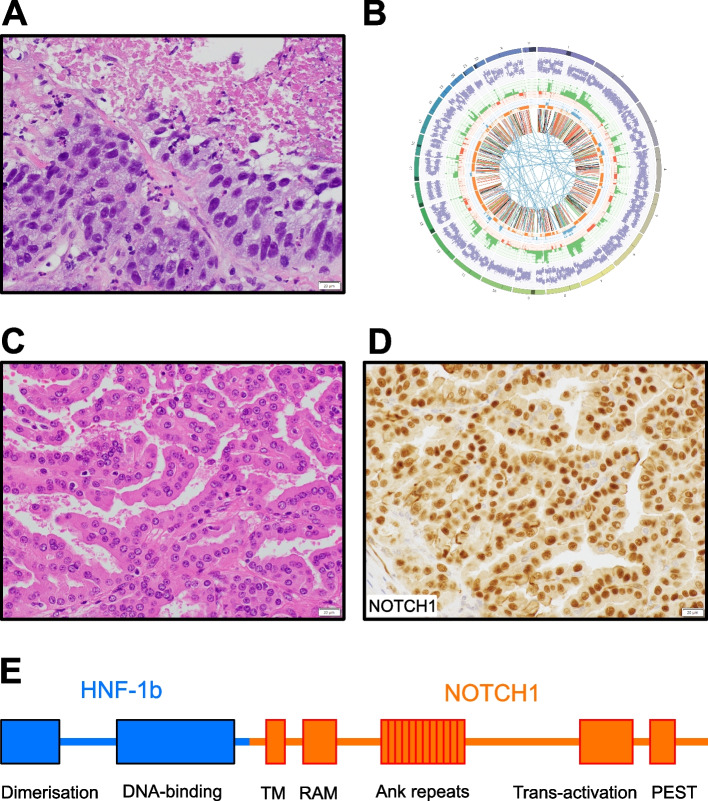
Case studies illustrating the potential utility of WGTS beyond diagnosis **A** Case 38 in the series. Micrograph of H&E-stained slide shows a pleomorphic epithelioid malignancy. This patient was a 31-year-old female with a new-onset right breast mass. Core biopsy revealed an undifferentiated, high grade malignant neoplasm, with a differential diagnosis including metaplastic (triple negative) breast carcinoma, malignant phyllodes tumor, undifferentiated sarcoma and undifferentiated melanoma. **B **WGS demonstrated a pathogenic germline BRCA1 splice site variant (ENST00000471181.7:c.70_80del) and somatic loss of heterozygosity. WTS detected aberrant BRCA1 splicing. A dominant Cosmic signature 3 was detected, [32] and the tumor was homologous recombination deficient, as determined by the HRDetect (score 0.88) and CHORD (76%) tools [34, 35]. Other somatic findings included a TP53 hotspot mutation (ENST00000269305.9 c.451C>T p.P151S) and MYC amplification (21 copies). Based on these findings, a confident diagnosis of metaplastic (triple negative) breast carcinoma could be made. Tumor/normal WGS data are represented as a Circos plot. Refer to Figure 5 for legend. Note the high number of red lines in the inner circle representing deletions characteristic of BRCA1-deficient breast cancer genomes. **C** Case 8 in the series. A 31-year-old female presented with a 32mm renal mass. Partial nephrectomy was performed, revealing a renal cell carcinoma with papillary architecture and oncocytic cytomorphology, for which classification was difficult. Micrograph of H&E-stained slide shows a renal epithelioid malignancy. **D** Nuclear expression of NOTCH1 in tumor demonstrated by immunohistochemistry. **E** WGTS detected an HNF1B::NOTCH1 fusion gene involving HNF1B exons 1-2 fused to NOTCH1 exons 28 onward, predicting a chimeric protein (left) containing the dimerization and POU DNA-binding domains of HNF1B and the transmembrane (TM) and intracellular domains of NOTCH1 (including the RBP-Jkappa-associated module (RAM), Ankyrin (Ank) repeats, transactivation and PEST domains). This fusion gene has been reported in a single papillary renal cell carcinoma case (TCGA/www.tumorfusions.org), [48, 64] leading us to postulate that this may represent a rare subtype of papillary renal cell carcinoma

An example of potential clinical utility beyond diagnosis is illustrated in Fig. 6a, b (Case 38).

In addition to clinical utility, the comprehensive nature of WGTS allows discovery of new and rare genomic features. We unexpectedly detected a *ZCCHC8::ROS1* gene fusion in a spindle cell rhabdomyosarcoma (case 43), a *ZFP64::NCOA2* gene fusion in an undifferentiated round-spindle cell sarcoma favored to be a mesenchymal chondrosarcoma, rather than a spindle cell rhabdomyosarcoma[44] in the absence of expression of skeletal muscle markers (case 6), a *SMARCB1-*deficient cutaneous squamous cell carcinoma (case 44)[45], a peritoneal mesothelioma with a *MAP3K8* rearrangement (case 20)[46, 47] and a renal cell carcinoma carrying a rare *HNF1B::NOTCH1* fusion (case 8; Fig. 6c–e)[48]. Overall, 11% of cases in our series were diagnosed as tumor (sub-)types that were either new or had only been described in small studies.

## Discussion

In this study, WGTS allowed a diagnosis to be established in 78% of patients with “difficult-to-diagnose” tumors. Owing to its histopathologist-initiated nature, and in contrast to focused cohorts such as sarcoma and CUP, our study supports broadening the scope of tumor/normal WGTS to resolve diagnostic dilemmas in other settings, including tumors of ambiguous histology, lineage, or malignant potential.

Our study’s premise differs from that of CUP, where tumors are favored to be metastatic by definition and of epithelial origin (95%, with only 5% unclassifiable as undifferentiated malignancies)[49]. In contrast, in our study, most tumors prior to WGTS were considered likely to be primary rather than metastatic (93%). Post-WGTS, our study revealed a much smaller proportion of epithelial malignancies (33%). Tumors in our study were frequently undifferentiated (53%) and/or of unknown lineage (56%), despite a work-up at least as thorough as that recommended for CUP[49]. The distinct clinical and histopathological characteristics of the “difficult-to-diagnose” tumors in our study support our model, whereby pathologists, in collaboration with genomic scientists, are best suited to determine the most appropriate diagnostic test, including WGTS. In CUP cases, pathologists typically define tumor lineage (e.g., adenocarcinoma or squamous cell carcinoma) but immunohistochemistry and clinico-radiological correlation have failed to determine tissue origin. For “difficult-to-diagnose” tumors, however, the primary challenge lies in determining lineage or malignant potential rather than tissue of origin. As these determinations fall within the pathologist’s responsibility, they are best placed to assess whether extensive NGS could aid in diagnosis.

The inability of traditional histopathological techniques to establish a diagnosis in a subset of tumors results from a variety of factors, including (1) undifferentiated tumors, for which usual morphologic and immunophenotypic “clues” for diagnosis are absent, (2) novel tumor types or subtypes, for which routine diagnostic methods lag behind research tools, and (3) tumors for which routine diagnostic methods yield conflicting or discordant results owing to false negative or positive results. The integration of comprehensive molecular data provided by WGTS into the diagnostic algorithm resolves many of these problems.

Tumor classification is currently evolving rapidly, as molecular data are incorporated into existing morphology-based classification schemes. Tumors that appear undifferentiated with conventional histopathological techniques and may have previously been diagnosed using generic terms such as “undifferentiated malignancy” are now able to be classified by molecular findings; for example, we recognize that a subset of melanomas will lack immunohistochemical expression of standard melanocytic markers[5, 50]. Among our cohort, there were several examples of undifferentiated malignancies for which molecular testing allowed refinement of diagnosis, including a metastatic cutaneous spindle cell squamous cell carcinoma (case 12), a dedifferentiated chondrosarcoma (case 10), and a metaplastic breast carcinoma (case 38). Even when diagnostic certainty was not attained, information provided by WGTS assisted in clinically relevant narrowing of the differential diagnosis in most cases. For example, a patient with an undifferentiated, pleomorphic malignant neoplasm with a differential diagnosis including undifferentiated melanoma, sarcomatoid carcinoma, or undifferentiated (pleomorphic) sarcoma, may benefit therapeutically if even one of these diagnostic possibilities can be eliminated.

WGTS allows recognition of novel or rare tumor types or subtypes, which conventional testing is unable to detect, as there is an inevitable lag in development of standard histopathological tests (such as IHC, FISH, and targeted NGS panels). Our cohort included cases for which a putative diagnosis of a novel or near-novel tumor type or subtype was rendered, while other cases representing examples of tumors seldom encountered in daily practice, several of which have been published as case reports[51, 52]. Reporting of these unusual cases contributes to the global understanding of tumor classification, as well as education. For this cohort of patients, establishing a diagnosis may provide some certainty to the patient, even if little data exists regarding optimal management[53].

In some cases, a diagnosis was suspected based on tumor morphology but was unable to be confirmed with conventional diagnostic techniques. WGTS allowed confirmation of the suspected diagnosis in some of these cases, including a *CIC*-rearranged sarcoma (case 3, in which *CIC *break-apart FISH was negative, as reported in a significant subset of cases)[54], and a low-grade fibromyxoid sarcoma (case 41), in which the diagnostic *FUS::CREB3L2* fusion had been unable to be demonstrated. In this study, WGTS also allowed us to confirm 4/4 tumors as recurrent but now undifferentiated malignancies. This highlights the difficulty in making such a diagnosis and suggests that pathologists should maintain a high degree of suspicion for a recurrence when confronted by an undifferentiated tumor in the context of a previous malignancy.

The benefit of WGTS in identifying potential therapeutic targets has previously been demonstrated[15–17] and although not the focus of this study, potentially actionable therapeutic targets were identified in 47% of our cohort. In a patient cohort comprising difficult to diagnose tumors, for whom standard clinical management pathways may be difficult to apply, identification of existing or clinical trial therapeutic targets is likely to be valuable[3, 10, 13, 14]. Pathogenic germline cancer predisposition variants were present in 9% of our cohort, similar to rates reported in other cohorts[55, 56]. Furthermore, resolution of diagnosis may have implications for understanding the patient’s prognosis, and impact surveillance, or suggest benefit for adjuvant therapy, or potential therapy in the setting of advanced disease. In our cohort, review by a medical oncologist suggested potential clinical implications beyond diagnosis in 67% of our patients. This study did not include extended clinical follow-up of the enrolled patients, which would provide more robust measures of true clinical benefit to this approach. Even for the subset of patients without identifiable “targetable” alterations or for whom management is unaltered, accurate diagnosis has intangible benefits, including providing understanding of disease for patients, clinicians, and authorities involved in health resource distribution.

Compared with other NGS assays, WGTS has both benefits and drawbacks as a diagnostic tool. Benefits include a near universal single test for identifying all tumor-related genomic variants, including those not targeted by panel assays, as well as novel genomic features. Thus, WGTS could reduce or eliminate the need to perform multiple smaller NGS tests (either sequentially or in parallel) and thereby reduce the associated costs of validating and maintaining these assays. However, despite these potential benefits, implementation of widespread WGTS of tumors is currently challenging. In part, this is due to input sample requirements: (a) In comparison to more widely available panel NGS tests, WGTS requires 5–20 × greater quantities of input material; (b) The theoretical sensitivity of WGS at a sequencing depth of 100 × is lower than that of panel-based NGS (depth typically 100–1000s-fold), predicting lower success rates on low-purity samples. However, in practice, a recent study demonstrated 100% concordance in SNVs and small indels between WGS and panel-based NGS on a set of 71 tumor samples despite low tumor cellularity (< 30%) in a third of cases[24]; c) The standard formalin fixation process in most pathology services compromises nucleic acid quality[57, 58]. This is a well-documented obstacle particularly for accurate detection of copy number and structural variants by WGS, which can be addressed by selection of suitable samples through DNA fragmentation assessment prior to WGS[24, 59, 60]. In addition to input sample requirements, financial costs, appropriate patient consent for potential germline findings, curation expertise, and clinically meaningful turn-around times (median 11 days has been reported[15]) are currently limiting factors. Currently, WGTS is available only in specialized centers able to support the test complexity. Nevertheless, since these difficult to diagnose cases are likely to be sent to large centers for review, it is conceivable that a network model could be developed to support laboratories unable to provide such complex testing. Additionally, with future developments, including decreasing sequencing and reporting costs, the benefits of WGTS may offset the potential costs associated with an uncertain diagnosis and potentially inappropriate clinical management.

In a subset of our cases diagnosis could potentially have been resolved by less complex testing, such as large panel NGS, which may be an option in settings where WGTS is not available. However, in 9/35 (26%) of those cases where WGTS informed diagnosis, no alternative test could be identified that would have been able to determine the diagnostically critical findings. These included mutations in genes that are not covered by commonly used NGS panels and alterations such as structural rearrangements that are not detectable by panel NGS. Furthermore, in contrast to more bespoke tests, WGTS collects negative findings across the genome, which can be informative, as we have demonstrated, particularly in the context of establishing a diagnosis of a novel or near-novel tumor type (such as case 8, illustrated in Fig. 6c–e). In addition, WGTS allows confident detection of genome-scale features such as mutational signatures, chromosome copy number changes, and near-haploidization, which can inform diagnosis, as illustrated by several cases in our cohort. For example, near-haploidization was a useful diagnostic clue in establishing a diagnosis of inflammatory leiomyosarcoma/inflammatory rhabdomyoblastic tumor (case 1, Additional file 1: Fig S2), while the presence of a UV-signature assisted in establishing a diagnosis in case 44 (a *SMARCB1*-deficient cutaneous squamous cell carcinoma).

In addition, WGTS can be used to run CUPPA, which was concordant with diagnosis of 17 cases. In 5 non-diagnostic cases, a high-confidence CUPPA prediction was obtained, matching one of the differential diagnoses. For the purpose of this study, we conservatively opted not to rely on CUPPA alone to determine diagnosis. Future work will establish how to weigh high-confidence CUPPA predictions in the diagnostic process. Conversely, CUPPA generated no matches in 15 cases in our cohort (33%). We note the large proportion of rare and mesenchymal tumors in our cohort, which are under-represented in the CUPPA training cohort. Thus, these results are in keeping with the design of CUPPA to produce low scores when assessing tumor types not represented in the training cohort. As the diversity of the training cohort grows, we anticipate CUPPA performance will further improve.

The use of WGTS led us to adapt our clinical practice and approach over the course of our study. Firstly, we developed and implemented a process whereby, where possible, we stored samples of fresh tumor tissue in media, in order to better preserve samples for potential WGTS. As another example, we noted that oncogenic gene fusions were enriched in the monomorphic category. Consequently, we implemented a targeted NGS RNA fusion panel for this category (this assay was not available at the beginning of this study). Nevertheless, despite this, there remains a subset of fusion-driven tumors for which WGTS maintains diagnostic advantages through full coverage (in comparison with targeted NGS panels) and the ability to compare DNA and RNA data.

Our cohort did include several cases for which diagnosis could potentially have been resolved by relatively simple testing, including one case of a fumarate hydratase-deficient renal cell carcinoma, for which immunohistochemistry for fumarate hydratase could have resolved the diagnosis (case 4). Such testing would now be considered standard of care testing at our institution, but this was not the case at the time of testing. The rapid increase in knowledge occurring as molecular testing becomes more widespread is necessarily impacting tumor classification, and diagnostic methods invariably lag behind and thus we believe that WGTS can provide an accelerated avenue to bridge that gap in challenging cases.

Our series likely displays institutional bias in that the cases derived from a quaternary referral center for adult patients, and there is under-representation of some tumor types, such as those from the central nervous system and female genital tract. Nevertheless, we suspect that similar diagnostic dilemmas are likely to be confronted at other institutions and that other diagnostic challenges may also be resolved with this approach. The cases included in this study constituted only a small minority of cases reported at a quaternary referral center (less than 1%), but as discussed above, the benefits are significant for this cohort and extend beyond tumor classification. We anticipate that the breadth of cases for which WGTS is useful is likely to change over time, as testing modalities change and tumor classification and therapeutic options evolve.

We also acknowledge that there is inherent subjectivity in the histopathological assessment of what constitutes a “diagnostic dilemma,” as well as the degree of certainty required to establish a diagnosis; both decisions will likely vary between pathologists and institutions, as well as over time. However, we believe that the fundamental principles are likely to be applicable broadly across institutions and would shorten the time to diagnosis.

## Conclusions

This pilot study has shown high clinical utility for pathologist determinable WGTS in a series of difficult to diagnose tumors. Funding and access to this test are not available for most patients. In Australia, there is limited reimbursement for molecular techniques for diagnostics. This contrasts with the UK genomic test directory, where there is widespread access to high-complexity cancer genomic testing, including WGS[61]. Indeed, in only 9 cases would the testing have been reimbursed under current Australian funding rules (i.e., testing covered by an item in the Medicare Benefits Schedule). Notably, the process for approving new test funding in Australia is arduous and lengthy. Future studies to define the subset of cases likely to benefit from WGTS and to collect data regarding clinical benefit may help to support such approval.

## Supplementary Information

Additional file 1: Supplementary figures 1-3

Additional file 2: Supplementary Table 1. Summary of all cases in the cohort

Additional file 3: Supplementary Table 2. Genetic alterations and relevant associated WTS observations reported in cases in the cohort

Additional file 4: Supplementary Table 3 Summary of Chi-square test of independence to determine if outcomes were correlated with age <50-years-old, or monomorphic versus pleomorphic morphology

## Data Availability

The WGS and WTS datasets supporting the conclusions of this article are available in the EGA repository under identifier EGAD00001015615 at https://ega-archive.org/datasets/EGAD00001015615 [62]. Data requests will be assessed by a Data Access Committee and granted to research studies that can demonstrate approval from a Human Research Ethics Committee (HREC) or comparable committee. This study did not generate new unique reagents.

## Abbreviations

CCGCM: Collaborative Centre for Genomic Cancer Medicine
COLUMN-PI: Cancer of Low survival and UnMet Need – Pathologist Initiated
CUP: Cancer of unknown primary
CUPPA: Cancer of Unknown Primary Prediction Algorithm
DRAGEN: Dynamic read analysis for genomics
EGA: European Genome-Phenome Archive
FFPE: Formalin Fixed Paraffin Embedded
FISH: Fluorescence in-situ hybridization
H&E: Hematoxylin and eosin
HRD: Homologous recombination deficiency
HREC: Human Research Ethics Committee
IHC: Immunohistochemistry
LOH: Loss of heterozygosity
MBS: Medicare Benefits Schedule
MPNST: Malignant Peripheral Nerve Sheath Tumor
NCCN: National Comprehensive Cancer Network
NGS: Next-generation sequencing
NHMRC: National Health and Medical Research Council
QC: Quality control
REN: Renal epithelial neoplasm
SNV: Single-Nucleotide Variant
SWI/SNF: SWItch/Sucrose Non-Fermentable
TM: Transmembrane
TMB: Tumor mutational burden
UK: United Kingdom
USA: United States of America
UV: Ultraviolet
WGS: Whole genome sequencing
WGTS: Whole genome transcriptome sequencing
WHO: World Health Organization
WTS: Whole transcriptome sequencing
