# Supplementary material for: Pathologist-initiated whole genome and transcriptome sequencing demonstrates diagnostic utility in resolving difficult-to-diagnose tumors
Source: Genome Med. 2025 Oct 7;17:107. doi: 10.1186/s13073-025-01534-5 (PMC12502407; doi:10.1186/s13073-025-01534-5)
Supplement: Supplementary file 1 — Additional file 1: Supplementary figures 1-3 [file 13073_2025_1534_MOESM1_ESM.pdf]

Figure S1

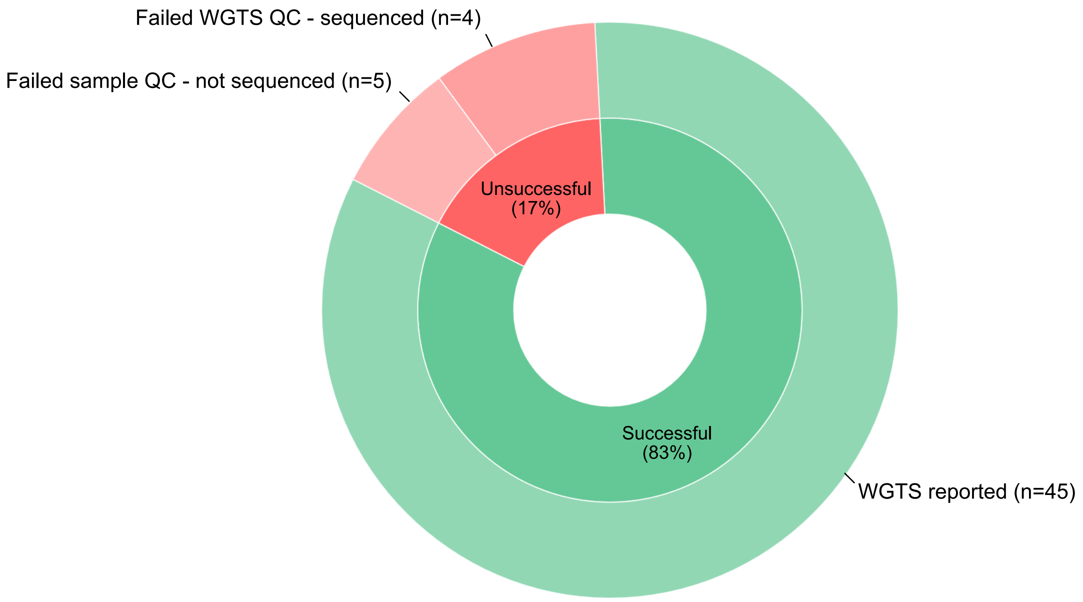

Supplementary Figure 1. WGS technical performance success rate.

Among 54 cases, WGTS was successful in 45 cases (83%) and failed in 9 cases due to either DNA (5 cases) or WGS (4 cases) quality control (QC) issues.

Figure S2

A

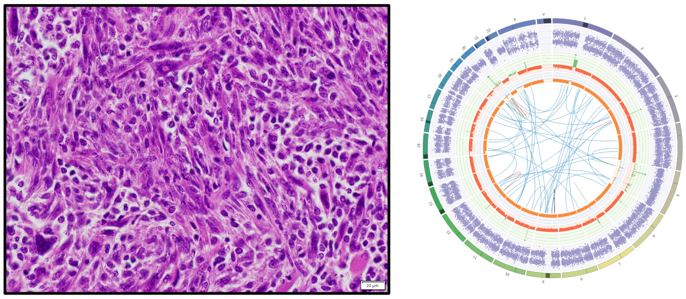

B

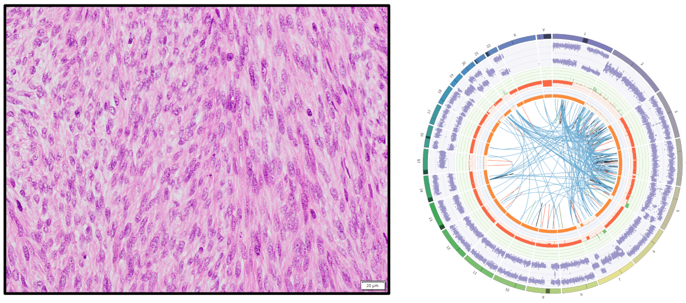

C

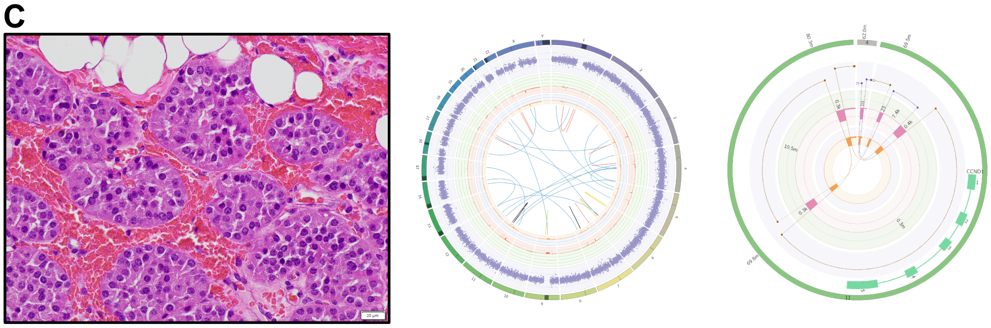

Supplementary Figure 2. Case studies.

(A, B) Example cases where tumor genome near-haploidization was diagnostically informative.

(A) Case 1. (left) Micrograph of H&E-stained slide showing a pleomorphic malignancy. Pre-WGTS diagnostic differentials: Inflammatory leiomyosarcoma (inflammatory rhabdomyoblastic tumor), metaplastic carcinoma and inflammatory myofibroblastic tumor. (right) Circos plot demonstrating near-haploidization sparing chr5, chr20 and chr22. For this analysis, a maximum tumor purity of 50% was applied. In addition, an *NF1* truncating mutation was detected. Features in line with inflammatory leiomyosarcoma.[1]

(B) Case 10. (left) Micrograph of H&E-stained slide shows a monomorphic spindle cell neoplasm. Pre-WGTS diagnostic differentials: Metastatic dedifferentiated chondrosarcoma (noting previous history of chondrosarcoma), monophasic synovial sarcoma, sarcomatoid mesothelioma, sarcomatoid carcinoma and undifferentiated melanoma. (right) Circos plot demonstrating near-haploidization sparing (parts of) chromosomes 2, 5, 7, 15, 19 and 21. Similar patterns of chromosomal loss have been observed in chondrosarcoma, localized and diffuse mesothelioma, and MPNST.[2-6] In addition, an *EED* splice site mutation (aberrant splicing confirmed by WTS) and homozygous deletion of *NF2*, *CDKN2A* and *MTAP* were detected. Features in line with dedifferentiated chondrosarcoma.[2,3]

(C) Case 35. Example of a benign tumor. (left) Micrograph of H&E-stained slide shows renal epithelial neoplasm. Pre-WGTS diagnostic differentials: renal oncocytoma (with renal vein, renal sinus and perinephric fat invasion), clear cell renal cell carcinoma and chromophobe renal cell carcinoma. (middle) Circos plot demonstrating a diploid tumor genome. (right) LINX plot[7] demonstrating complex rearrangement of chr11q13 with breakpoints surrounding *CCND1*, in keeping with a renal oncocytoma.[8,9] Track 1: Chromosomes. Track 2: Gene (*CCND1*). Lines: structural variants chained into two continuous predicted derivative chromosomes. Track 3: copy number gains (green) and losses (purple). Track 4: Minor allele copy number gains (blue) and losses (orange).

Fig S2, continued

Supplementary references

1. Arbajian et al. (2018) Mod Pathol *31*, 93-100. 10.1038/modpathol.2017.113.
2. Olsson et al. (2011) PLoS One *6*, e24977. 10.1371/journal.pone.0024977.
3. Cross et al. (2022) Genome Med *14*, 99. 10.1186/s13073-022-01084-0.
4. Hung et al (2020) Mod Pathol *33*, 271-280. 10.1038/s41379-019-0330-9.
5. Hung et al (2020) Mod Pathol *33*, 2269-2279. 10.1038/s41379-020-0588-y.
6. Sukov et al (2010) Cancer Genet Cytogenet *202*, 123-128. 10.1016/j.cancergencyto.2010.07.120.
7. Shale, C. et al (2022) Cell Genom *2*, 100112. 10.1016/j.xgen.2022.100112.
8. Sukov et al (2009) Hum Pathol *40*, 1296-1303. 10.1016/j.humpath.2009.01.016.
9. Joshi et al (2015) Cell Rep *13*, 1895-1908. 10.1016/j.celrep.2015.10.059

Figure S3 Clinical implications

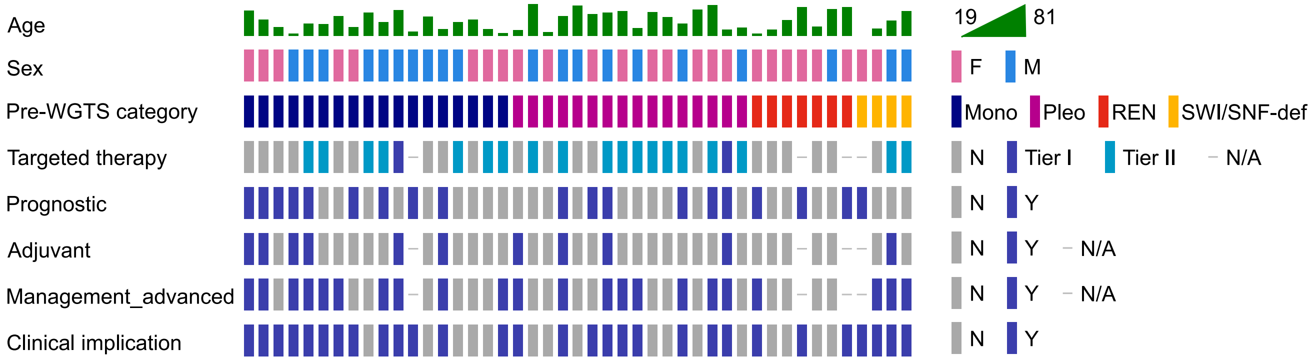

Supplementary Figure 3 Theoretical clinical implications beyond diagnosis

Legend:

Sex: F – female; M – male. Pre-WGTS category: Mono – monomorphic; Pleo – pleomorphic; REN – Renal Epithelial Neoplasm; SWI/SNF-def – *SMARCB1* or *SMARCA4* deficient. Fusion: fusion gene detected. Targeted therapy: N – no therapeutic target identified; Tier I – Target for guideline-recommended, reimbursed treatment identified; Tier II – genetic alteration satisfying clinical trial entry criteria identified; N/A – not applicable owing to diagnosis of benign neoplasm. Prognostic: WGTS significantly informed prognosis (retrospective analysis). Adjuvant: WGTS significantly informed therapy selection in the adjuvant treatment setting (retrospective analysis). Management_advanced: WGTS significantly informed therapy selection in the advanced disease setting (retrospective analysis). Clinical implication beyond diagnosis: Summary of WGTS findings informing prognosis, adjuvant treatment and advanced disease management. N – No; Y – Yes; N/A – not applicable due to diagnosis of benign neoplasm.
